# Primary symptoms of severe mycoplasma pneumoniae pneumonia with acute abdomen, scrotal swelling and pain, and fever: A case report

**DOI:** 10.1002/iid3.955

**Published:** 2023-10-03

**Authors:** Hui Lei, Qiong Wang, Xue‐min Xiong, Shengjuan Hu, Yu Shao, Rongchang Shao

**Affiliations:** ^1^ Department of pediatrics Ezhou Central Hospital Ezhou Hubei China

**Keywords:** abdominal pain, fever, mycoplasma pneumoniae pneumonia, scrotal swelling and pain

## Abstract

**Background Introduction:**

In recent years, there has been an increase in the number of patients diagnosed with pediatric diseases who have severe *Mycoplasma pneumoniae* (MP) pneumonia, and there has also been an increased attention to serious extrapulmonary complications. However, cases with abdominal pain, acute abdomen, scrotal swelling and pain, and fever as the primary symptoms have been rarely reported.

**Case Description:**

A 3‐years‐and‐8‐months‐old male patient diagnosed with pediatric disease was reported with abdominal pain, scrotal swelling and pain, and fever as the primary symptoms in the present study. No respiratory symptoms were observed throughout the disease. Through computed tomography (CT) scanning, the patient was diagnosed with severe MP pneumonia based on the symptoms of abdominal pain and fever, as well as pulmonary infection, pleural effusion, and retroperitoneal exudation. Laboratory tests supported the diagnosis of MP infection, and the diagnosis was confirmed by severe MP pneumonia. The *therapeutic* effects of azithromycin were poor, and the symptoms were quickly alleviated with the addition of gamma globulin and methylprednisolone. After discharge, azithromycin sequential therapy was administered. The chest CT was normal at the follow‐up 1‐month later.

**Conclusion:**

Severe MP pneumonia in patients with pediatric diseases may include abdominal pain, scrotal swelling and pain, and fever as the primary symptoms. Care should be taken to avoid missed diagnoses and misdiagnoses in clinical practice.

## Graphical abstract

Severe *Mycoplasma pneumoniae* pneumonia in pediatric patients might present with abdominal pain, scrotal swelling and pain, and fever as the primary symptoms. Attention should be paid to avoiding missed diagnoses and misdiagnoses in clinical practice.

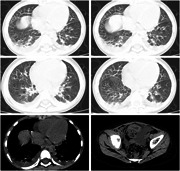

## 1 INTRODUCTION

*Mycoplasma pneumoniae* (MP) is a cell‐wall‐free, self‐replicating, minimal prokaryotic microorganism that can invade the respiratory epithelium and cause respiratory diseases such as upper respiratory tract infection, bronchiolitis, bronchitis, and pneumonia.^1^ Approximately 1/3rd of cases was asymptomatic, in the form of tracheobronchitis, pneumonia, and tympanitis as the most severe pneumonia. MP pneumonia with acute abdomen, scrotal swelling and pain, and fever pains as the primary symptoms and without obvious respiratory symptoms has not been reported; therefore, missed diagnoses and misdiagnoses are possible. In the present study, we reported a patient with severe MP pneumonia with acute abdomen, scrotal swelling and pain, and fever as the primary symptoms admitted to the Ezhou Central Hospital in May 2021 in an effort to improve the understanding of the disease among pediatricians and reduce the incidence of misdiagnoses and missed diagnoses.

### 1.1 Clinical material

A 3‐years‐and‐8‐months‐old male patient was admitted to the hospital in May 2021 with 4 days of fever and abdominal pain. The peak body temperature was 39.5°C. The patient had intermittent abdominal and scrotal pain, but no vomiting was observed. The stool, which was passed 1−2 times per day, was smooth and showed signs of loose stools. No cough, wheezing, shortness of breath, or other symptoms were present. The patient was in good health, had normal physical and mental development, and had no other unusual medical history besides upper respiratory tract infection (1−2 times per year on average). All family members are healthy and there is no history of genetic disease in the family; the patient is a preschooler who receives a normal preschool education and has no mental disease.

**Figure 1:**
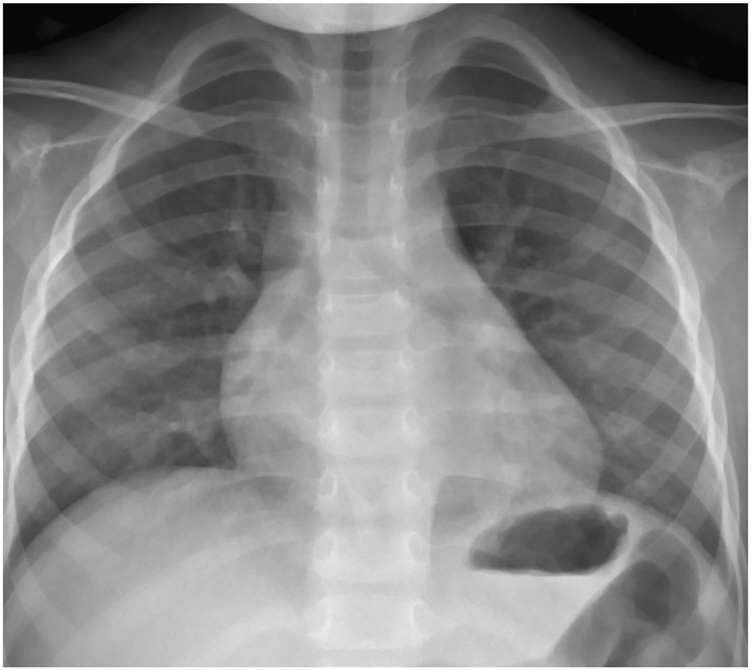
The chest X‐ray at admission in the pediatric patient, showing no obvious abnormalities in the chest and abdomen.

**Figure 2:**
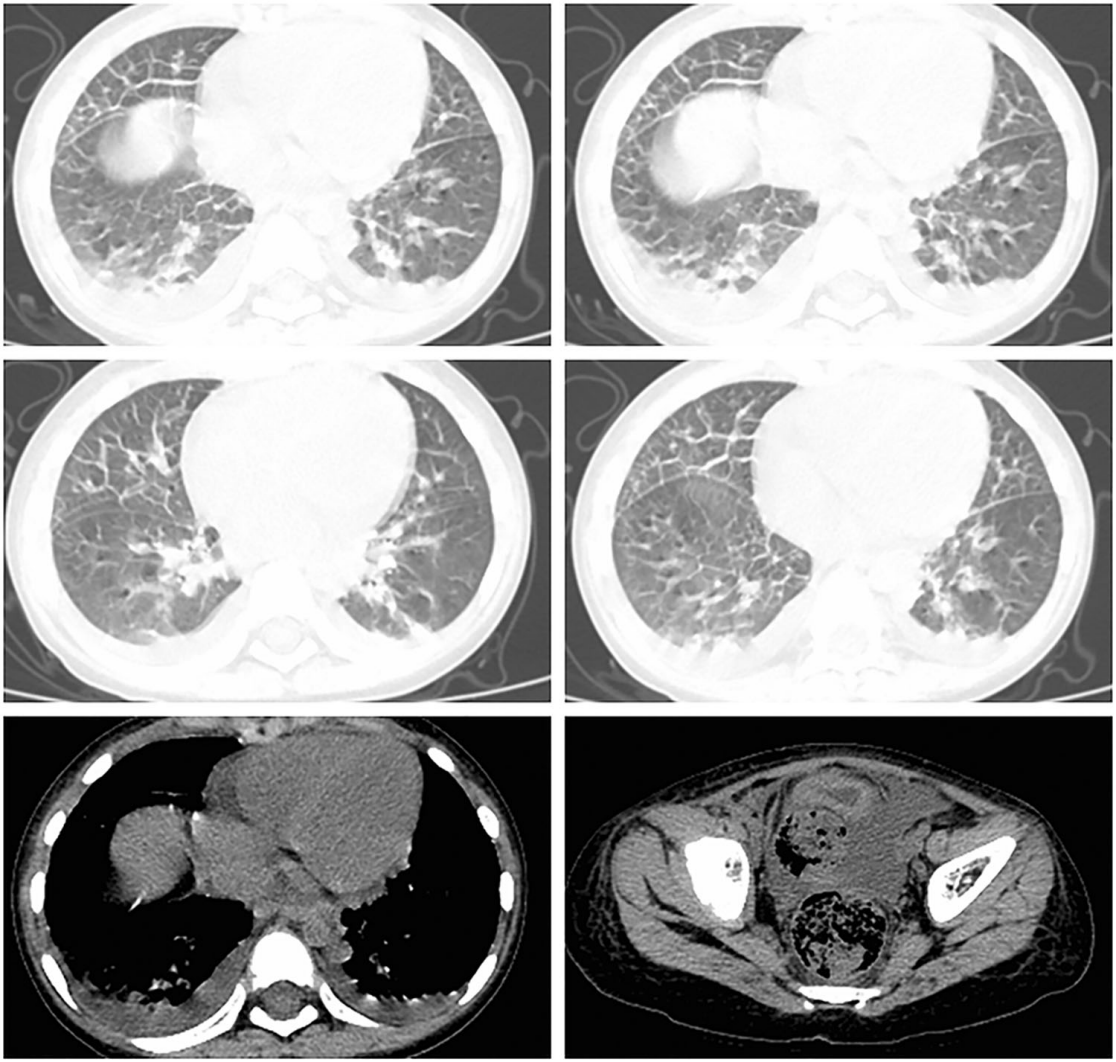
The chest and abdominal CT after admission in the pediatric patient, results are as follows: 1. infectious lesions in the lower lobes of the bilateral lungs, bilateral pleural effusion; 2. considerable stagnation of the contents of the colorectal cavity; 3. a little flocculent osmosis in the retroperitoneum, and the possibility of infectious diseases could not be excluded; 4. pelvic effusion. CT, computed tomography.

**Figure 3:**
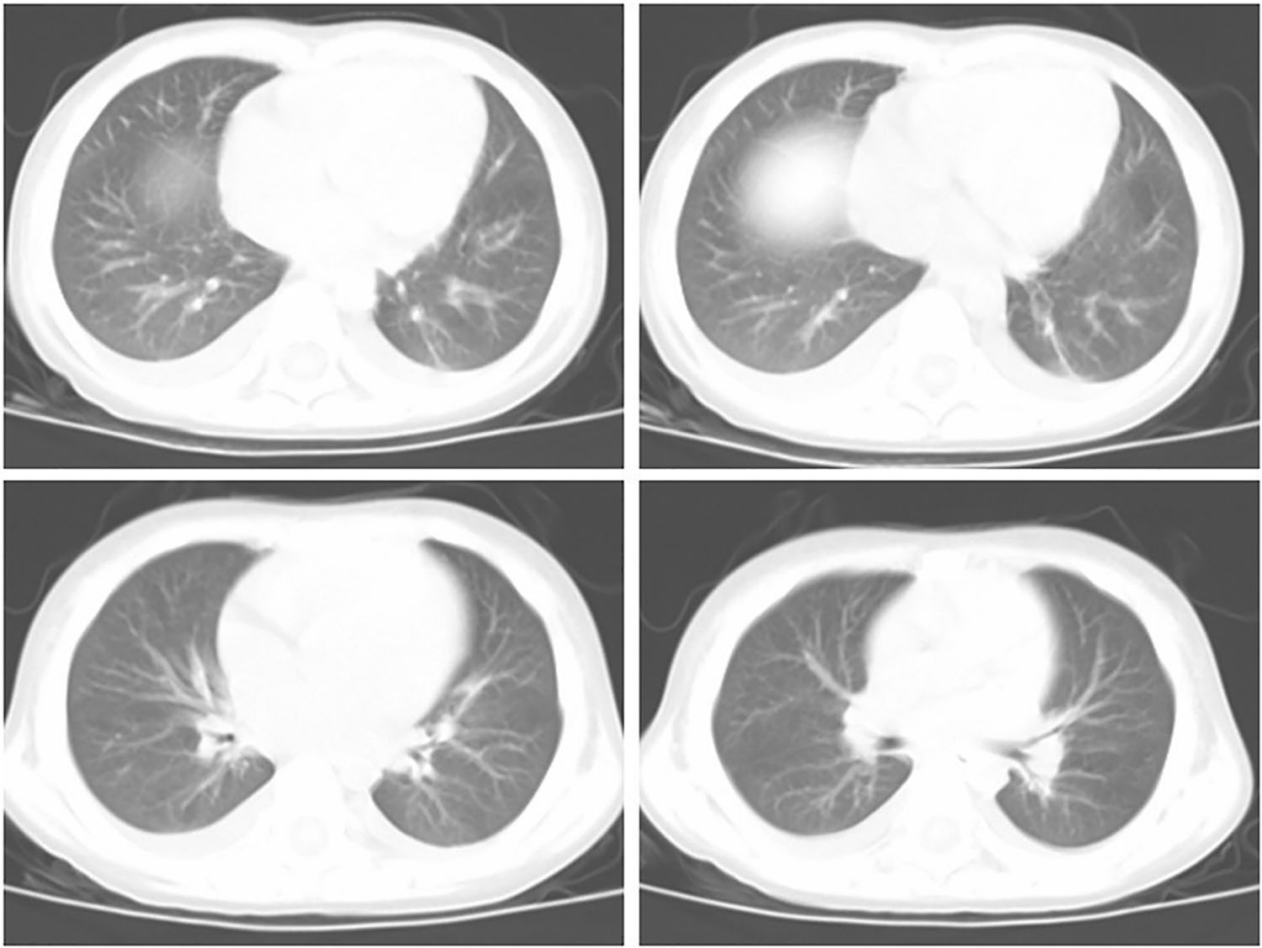
The review of chest CT after treatment in the pediatric patient. The bilateral lung lesions and pleural effusion were significantly absorbed and reduced, and the abdominal CT revealed no abnormalities. CT, computed tomography.

History: The patient with pediatric disease had a history of eczema and urticaria‐related hospitalizations. Physical examination results at admission: The patient's temperature was 39°C, his pulse rate was 120 beats/minute (normal), and his respiration rate was 22 times/minute (regular). The patient was conscious and alert. The neck was soft and there was no obvious rash on the skin or mucous membranes anywhere on the body. The bilateral breath sounds were audible, and neither dry nor wet rales were evident. The heart sounds were strong and regular without obvious murmurs. The abdominal muscles were tense and experiencing pain around the umbilicus. The patient with pediatric disease refused pressure to the abdomen. There was no obvious mass palpable in the abdomen. The skin of the scrotum was red, swollen, and extremely tender. The bowel sounds weakened slightly. The muscle strength of the limbs was normal with negative pathological signs. The results of the laboratory examinations were as follows (Table 1). The hepatic and renal functions, electrolytes, amylase, and lipase were normal. A plain X‐ray of the chest and abdomen found no obvious abnormalities (Figure 1). A color Doppler ultrasonography of the abdomen as well as of the scrotum and epididymis revealed no obvious abnormalities. The tests for antituberculosis antibodies were negative as were the results of the combined tests for autoimmune antibodies (−). After admission, ceftizoxime (0.75q every 12 h) was administered intravenously and azithromycin (0.15qd daily) orally for 3 days. The pediatric patient continued to have repeated fever and abdominal pain. The abdominal muscle was tense, and the patient refused any pressure on the abdomen. There was no obvious relief to the red and swollen scrotum or tenderness. Results of the routine blood test is in Table 1; the MP antibody IgM was a strong positive, and the MP antibody IgG was negative. The results of a plain CT scan and three‐dimensional reconstruction of the chest, abdomen, and pelvis were as follows: 1. Infectious lesions in the bilateral lower lobes of the lungs and bilateral pleural effusion; 2. significant stagnation of the colorectal cavity's contents; 3. With a small amount of flocculent osmosis in the retroperitoneum, infectious diseases could not be ruled out; 4. pelvic effusion (Figure 2). Also, Widal's reaction, tuberculosis TSPOT, blood culture, and sputum culture were negative. The oral administration of azithromycin was continued, and gamma globulin was administered for 3 days (400 mg/kg.day) together with methylprednisolone for 3 days (2 mg/kg.day). Body temperature was maintained for 24 h following administration, and the symptoms of abdominal pain, abdominal muscle tension, scrotal redness, and tenderness were significantly alleviated. These symptoms disappeared after 48 h. Azithromycin was administered for 7 days. After 4 days, CRP and ferritin were normal, and the erythrocyte sedimentation rate (ESR) had decreased to 21 mm/h on review. Simultaneously, the respiratory pathogen combination was analyzed: MP‐IgM (strong positive) and MP‐IgG. (weak positive). The chest CT results revealed that the bilateral lung lesions and pleural effusion had been significantly absorbed and reduced (Figure 3), whereas the abdominal CT revealed no abnormalities. After discharge, azithromycin sequential therapy was administered orally for two cycles (with administration for 3 days and discontinuation for 4 days). The chest CT was normal at follow‐up 1 month later. Simultaneously, the following respiratory pathogen combinations were examined: MP‐IgM (negative) and MP‐IgG. (positive). No relapse had occurred at the time of the telephone follow‐up, 6 months later.

**Table 1:** Laboratory data of the patient.

	WBC (*10^9^/l)	N (%)	L (%)	HB (g/l)	PLT (*10^9^/l)	CRP (mg/l)	ESR (mm/h)	IL‐6 （pg/ml）	PCT （ng/ml）	MP‐IgM	MP‐IgG
Day 4	6.61	76	14.7	111	163	86.5	51	141.05	5.04	+/−	−
Day 8	4.66	63.9	21.8	83	126	8.75	27	179.32	2.0	+	−
Day 15	/	/	/	/	/	/	21	Normal	Normal	++	+/−
1 month after discharge	/	/	/	/	/	/	/	/	/	/	++

## 2 DISCUSSION

In the present case, the patient was presented with acute abdominal and scrotal swelling and pain as well as fever as the primary symptoms, and these symptoms became more prominent as the disease progressed. After a comprehensive physical examination and imaging examinations, the final diagnosis was confirmed; no pertinent clinical reports have been identified. It was suggested that the initial manifestation of MP pneumonia could occur outside of the lungs, and the manifestations could be extremely atypical; therefore, care should be taken in clinical practice to avoid misdiagnoses and missed diagnoses.

The pathogenesis of MP‐related extrapulmonary diseases might be divided into three types. (1) Direct injury: MP may release enzymes such as hydrolase, nuclease, and phosphatase into host cells to disrupt the normal cellular activities. MP may secrete numerous adhesins and adhesion proteins to adhere to cells, resulting in damage to the host cell membrane. MP is capable of invading host cells, consuming nutrients, and releasing toxins.^2^ In addition, MP may inhibit the activity of catalase in host cells, rendering them susceptible to hydrogen peroxide‐induced injury and necrosis and resulting in the proliferation of local inflammatory factors.^3^ (2) Indirect factors: MP may generate lipid‐related membrane proteins and activate toll‐like receptors, allowing MP to not only cause cell damage directly but also generate damage‐related molecules, which would induce inflammatory responses leading to cell damage.^4^ Therefore, MP may not only directly induce the inflammatory response at the site of occurrence, but also directly appear at the site of inflammation, with the induction of monocyte‐macrophages, lymphocytes, and airway epithelial cells, producing a variety of inflammatory factors and causing intrapulmonary and extrapulmonary injuries via excessive inflammatory responses. In addition, the presence of common antigens in the cytoplasm of MP and the human body may result in multiple organ injuries through a variety of immune complexes, which is currently regarded as the most probable pathogenesis of MP‐related extrapulmonary disease. (3) Vascular injury: MP may generate cytokines and chemokines, which may directly or indirectly result in blood flow obstruction.^5^ The glycolipids present on the surface of MP may cross‐react with cardiolipin on the surface of platelets and vascular endothelial cells, inducing the production of anti‐cardiolipin antibodies that result in vascular endothelial injury and systemic hypercoagulability, activating chemical mediators such as complement, and causing thrombotic vascular occlusion.

MP infection is typically accompanied by a variety of nonspecific gastrointestinal symptoms, most of which may have hepatic dysfunction.^6^ Kim et al. reported a rare gastrointestinal complication of acute pancreatitis after MP infection.^7^ In the present case, the patient experienced abdominal pain, abdominal muscle tension, and a refusal to accept pressure to the abdomen. The abdominal CT results revealed a small amount of flocculent exudation in the retroperitoneum, but the hepatic function was normal, with normal lipase and amylase. The abdominal B‐ultrasonography and CT did not reveal any pancreatic abnormalities, so hepatic dysfunction and pancreatitis were ruled out. With the administration of gamma globulin and glucocorticoids, abdominal pain, abdominal muscle tension, and scrotal swelling and pain were quickly alleviated, indicating that these symptoms were primarily caused by immune disorders caused by infection. In addition, pleuritic exudation could cause an inflammatory reaction involving the diaphragm and lower parietal pleura, which would be transmitted through the 7th–11th intercostal nerves around the peritoneum, resulting in referred pain around the umbilicus. The inflammatory exudation in the retroperitoneum would directly stimulate the nerves surrounding the peritoneum, causing excruciating pain; this could be misdiagnosed as acute abdomen.^8^ Zhang et al. reported a patient with pleurisy and unilateral pleural effusion and abdominal pain as the primary symptom.^9^ In the present case, fever and acute abdomen were the predominant symptoms; however, for these symptoms to be accompanied by pleural effusion and retroperitoneal flocculent exudation is extremely rare.

Another primary symptom in the present case is pain and scrotal swelling, which have not been previously reported until now. The skin lesions of MP infection are most common as rashes, including erythema, maculopapular rash, blisters, papules resembling measles‐like or scarlet fever, urticaria, purpura, and even Stevens‐Johnson syndrome.^10^ Jacobs et al. reported a case of priapism in a child with MP infection, which was correlated with vascular occlusion caused by MP infection.^11^ In the present case, the pathogenesis of scrotal swelling and pain remains unclear. From the rise in D‐dimer and the patient's response to treatment, this may be associated with immune disorders and vascular injury. These pathogeneses are comparable to those of the aforementioned cases. The limitation of this study is that, this is the only case to date, we will need to collect data from additional cases in the future.

In conclusion, with more in‐depth investigations into mycoplasma infection, we are becoming increasingly aware of the mechanism and pathogenesis of related diseases. As a result of this case, we have acquired a new understanding of MP infection and extrapulmonary diseases, which may help guide future clinical practice.

## AUTHOR CONTRIBUTIONS

*Conception and design of the research*: Hui Lei and Rongchang Shao. *Acquisition of data*: Qiong Wang and Xue‐min Xiong. *Analysis and interpretation of the data*: Hui Lei and Qiong Wang. *Statistical analysis*: Shengjuan Hu and Yu Shao. *Writing of the manuscript*: Hui Lei and Rongchang Shao. *Critical revision of the manuscript for intellectual content*: Qiong Wang and Rongchang Shao. All authors read and approved the final draft.

## CONFLICT OF INTEREST STATEMENT

The authors declare no conflict of interest.

## ETHICS STATEMENT

I confirm that I have read the Editorial Policy pages. This study was conducted with approval from the Ethics Committee of Ezhou Central Hospital. This study was conducted in accordance with the declaration of Helsinki. Written informed consent was obtained from all patient guardians. All patient guardians signed informed consent to publish detailed information about the case.

## Data Availability

All data generated or analyzed during this study are included in this article. Further enquiries can be directed to the corresponding author.
